# On the Use of Glycerine in Diseases of the Ear

**Published:** 1849-11

**Authors:** 


### ECLECTIC DEPARTMENT,

### AND SPIRIT OF THE MEDICAL PERIODICAL PRESS.

*On the Use of Glycerine in Diseases of the Ear.* By THOMAS H. WAKLEY, ESQ,. Surgeon to the Royal Free Hospital, London.

The anxiety which is always manifested by the public to take advantage of any new discovery or successful mode of treatment connected with the removal of disease, affords a strong proof of the confidence which is generally entertained by the community in the power of medicine as it is practised in this country· In this respect a marked difference is shown in the conduct of the professional and non-professional portion of society. This is not extraordinary, as afflicted persons naturally seek for the readiest mode of obtaining relief. The medical practitioner, on the contrary, first considers the rationality of any proposed method of effecting a cure. His studies have taught him to reflect and to reason, and the results of experience have admonished him not to form conclusions too hastily, or to generalize too widely from restricted data. But it must not be inferred, that because the medical practitioner is less prompt than the public in resorting to the aid of a new agent, that his confidence in the powers of the healing art is less forcible, or is less deeply fixed in the foundations of his judgment, than is that of the non-professional community; on the contrary, the confidence which he feels is not the offspring of an unreflecting, ill sustained faith—it is not a weak superstructure, raised upon the narrow basis of a single fact, but arises from, and is sustained by the broad and solid foundations of science, reason and experience. It is well for society and the progress of knowledge, that the minds of medical practitioners are thus trained and disciplined; for if it were a custom with them, without hesitation and reflection, to adopt and sanction every newly-announced successful plan of treating disease, the subsequent failures, in a great majority of the novel plans, would soon destroy all confidence in the practice of medicine, not only in the public mind, but amongst physicians and surgeons themselves. Probably one of the causes which has tended in a great degree to establish and sustain the confidence of the community in the practice of medicine, has been the judicious caution which practitioners of eminence have exercised before they have resorted to new means of cure; and secondly, the candour and integrity which they have so often displayed in publishing the results of their experiments and experience. It is honourably felt that much deliberation and caution are due alike to a noble science, to the just claims of society, and to the exalted character of a dignified profession. If every example of this successful treatment of disease, by a new remedy, were to be published, the minds of practitioners, if not strongly fortified by previous study, and habits of thoughtful investigation, would become in danger of being involved in confusion by the dazzling announcements of numberless triumphant experimentalists. It is, therefore, forcibly felt by the profession, that before a new mode of treating disease be recommended, something more substantial than speculation or hypothesis should be available to warrant its adoption. Reflections of this kind have induced me to pause for a very considerable period before I determined respectfully to submit to the consideration of the profession, the humble pretensions which I am desirous of establishing for *glycerine* in the treatment of certain forms of deafness. The strong conviction which I entertain as to the utility and value of this remedy is the only apology I can offer for claiming, even for a moment, the attention of the profession on such a subject. I have no new doctrine to inculcate; no new discovery in physiology to enforce; no "great fact" in pathology to disclose. The sum total of my aim on this occasion is, to contribute a fact to our therapeutic store, which, although it may be regarded by some as a very insignificant item, is one which I think ought to be very generally known. I have already adverted to the confidence felt by the public in the capabilities of medicine, as a practical science, and to the eagerness shown by society to take advantage of any newly-announced medical or surgical remedy. Unfortunately, that unsuspecting desire to seize, with blind faith and hope, on any new proposal for curing disease, is, in this country, the fertile source and support of all those partial, detached and empirical systems of practice which are, and ever must be denounced and repudiated by every well-educated practitioner.

The medical officers connected with the public institutions of this vast metropolis command the most ample opportunities of observing the strong tendencies of the public feeling with respect to the adoption of new remedies. Scarcely is a new fact of any importance connected with the cure of disease published in the periodical journals, when the "out-patients" at the public hospitals make the proposed remedy the subject of common conversation amongst them. Frequently the applicants for relief even ask to be treated on the "new plan," and sometimes they request to be given some of the new "medicine."

One of the most striking instances of the lively effect produced by the first announcement of a "new plan" of treatment, was afforded in the summer of last year, by the publication, in The Lancet, of the first of a series of valuable papers by Mr. Yearsley, entitled, "On a New Mode of Treating Deafness." Immediately after the appearance of those papers, an influx of a new class of patients was observed at the Royal Free Hospital. The members of that new class of persons were afflicted with deafness, and often was the remark made by them, "I wish, if you please, to be treated upon the new plan; or the question was asked, "if there had not been discovered a cure for deafness?" Such inquiries from patients suffering under actual disease, many of whom stated that they were deprived of the means of obtaining a livelihood, in consequence of their infirmities, suggested the questions—"What ought the surgeons of a public general hospital to do, under such circumstances?" "Ought these patients to be rejected at this place, and transferred to the institutions specially devoted to diseases of the ear?" It appeared to be just that the patients should be received.

Accordingly, I resolved to attempt to confer a benefit on the applicants, by adopting and following out the plan of treatment recommended by Mr. Yearsley; and this resolution was carried into practice, with results which rapidly increased the number of expectant patients. Would it have been right, I ask, to refer such patients to other institutions by rejecting their supplications for relief, or, worse still, to consign them possibly to a class of not over-scrupulous practitioners, who profess to work miracles, but fortunately whom medicine does not claim, or in any manner recognize as her legitimate children. Let it not be considered, from these remarks, that I deprecate a division of labor in the practice of medicine and surgery. On the contrary, I distinctly acknowledge, that an intense scientific application devoted to "specialities" in the investigation and treatment of disease, has been productive of highly useful and important results. At the same time, such divisions of labor cannot, in my opinion, justify the surgeon of a public hospital in neglecting the consideration and treatment of any class of diseases which properly fall within the field of observation. I need not dwell upon the importance of the sense of hearing. Why should a surgeon admit that a disease of the eye or the tongue properly demands his interference and best attention, and then altogether reject as unworthy of notice or consideration the deranged structures of the organ of hearing. The principles of diseased action are the same in all the vital organs, although peculiarities of tissue produce different morbid results.

During the existence of the first flush of success, the value of Mr. Yearsley's new method of treatment may have been over-estimated. This was not his fault; and the fact cannot be disputed, that Mr. Yearsley openly and candidly submitted his views to the notice of the profession. However, it happened that poor patients, in considerable numbers, claimed, at the portals of the public hospitals, to be recipients of the advantages of the new discovery, and I then thought, and still think, that it would have been ungracious and unfeeling to have rudely directed the sufferers to apply elsewhere for relief.

The adoption of the plan of treatment recommended by Mr. Yearsley was an appropriate recognition of the value of his labors. The success which attended the use of the simple operation which he recommended was, as I have already remarked, very striking in the first instance. In several cases, the effect of the application of the wetted cotton, in which the tympanum had been perforated by ulceration, was even extraordinary. But it too frequently happened, that the relief obtained was of an ephemeral duration. On applying the wetted cotton, the power of hearing, in several instances, which had been lost for a very long period, was instantaneously restored—an event which excited the most profound astonishment in the minds of patients and their friends. Too soon, however, was it perceived that the newly-acquired power gradually subsided, and the sense of hearing returned to its previous imperfect condition. The relapse frequently produced a feeling of dejection in the spirits and hopes of the patient, which it was painful to witness. The benefit derived from the application, in the first instance, was undoubted, and could not be mistaken; hence arose the question—Why was it of so evanescent a character? This inquiry naturally suggested a minute investigation into the nature of the materials employed, and also their immediate and remote influence on the parts to which they were applied. A brief investigation, a few experiments, and an attentive consideration of the subject, induced me to attribute all the conferred advantages to the effects produced by the water, and to reject the cotton as nearly, if not entirely useless. It even appeared that after the water had evaporated, the retained dry cotton became an additional impediment to the function of hearing. What was to be done? What were the indications which such facts seemed to establish? Evidently the use of some agent, which, by offering a successful resistance to evaporation, should retain its moisture, and continue to lubricate the auditory canal. Clearly enough, it was from the moisture that the benefit was obtained, and from a continuance of the moisture was the advantage to be prolonged. On duly considering all that I had observed, it appeared to me that *glycerine* was the only agent which was at all likely to accomplish the object I had in view. After consulting with Mr. Lloyd Bullock, of Conduit street, relative to the composition and properties of glycerine, my opinion as to the propriety of giving it a trial, was confirmed; and Mr. Bullock kindly manufactured for me a small quantity of that preparation in its purest form. This portion I obtained from Mr. Bullock in the first week of August, 1848, and employed it immediately in several cases, with apparently the most complete success. One of the patients, aged nineteen years, was a relative of Mr. Braithwaite, the celebrated engineer. In this instance the deafness had existed from infancy. Reports of four of these cases will be found amongst those attached to this paper. They were the first I treated with the new agent. In all these patients the wetted cotton had failed to produce a lasting benefit. Two of the four patients are now completely cured; and the other two are so far recovered as only to find it necessary to resort to the glycerine at distant intervals. The success of the new remedy, in these and many other instances, has attracted much notice; and I have now used the glycerine in upwards of three hundred cases of deafness. On many occasions it has been employed without any advantage whatever. In other instances the benefit was considerable for a short time, and then disappeared. In numerous cases, however, by the use of it, the power of hearing had been completely restored.

It was only after much experience in the application of glycerine, and from observing its action in a great number of cases, that it could be ascertained what were those conditions of the ear in which it was most likely to prove of advantage. Contrary to what might have been anticipated, the use of the remedy was successful in persons in whom the deafness had been of many years' duration—one, for example, thirty years, and also in cases where the existence of the malady could be traced to the eruptive fevers of childhood. In instances of deafness caused by inflammation, followed first by suppuration, and then by a horny, dry condition of the auditory canal, the application of glycerine has been attended with signal advantage. Equally marked and peculiar is the success when it is used in cases where there is a partial or total absence of ceruminous secretion. In many instances of deafness belonging to these classes of cases, the employment of glycerine has been followed by a perfect restoration of the power of hearing. In other examples of deafness, where the membrana tympani had evidently become thickened and hardened, and on examination with the speculum, denoted a whitish or pearly appearance, the use of the glycerine was followed by strikingly beneficial and gratifying effects. It is evident, therefore, that the application of glycerine is equally admissible, whether the tympanum be in a sound state, or whether it has been destroyed by ulceration.

A description of the composition and properties of glycerine, abridged from Turner's "Elements of Chemistry," may not be uninteresting on this occasion.

Glycerine was discovered by Scheele, and Chevreul proved its exact composition and constitution. Its formula is $C_6H_7O_5+aq$. It is found in fatty oils combined with oleic, stearic and margaric acids; its specific gravity is 1.252. Glycerine is a syrupy liquid, miscible both with alcohol and water, insoluble in ether, slightly inflammable, inodorous, and of a sweet taste.

The most convenient mode of preparing it is by the saponification of olive oil, by means of litharge and a little water. Sulphuric acid will separate the oily matters, leaving an aqueous solution containing the alkaline salt along with the glycerine. The mixture is evaporated to dryness, and treated with alcohol, which again dissolves the glycerine, and leaves the alkaline sulphate undissolved. The glycerine may be purified from oxide of lead, by passing through it a current of sulphuretted hydrogen.—*London Lancet.*

Appended to the foregoing are abstracts of several cases which we omit.

EDITOR.

